# Body composition in male lifelong trained strength, sprint and endurance athletes and healthy age-matched controls

**DOI:** 10.3389/fspor.2023.1295906

**Published:** 2023-10-31

**Authors:** Simon Walker, Mikaela von Bonsdorff, Sulin Cheng, Keijo Häkkinen, Dmitriy Bondarev, Ari Heinonen, Marko T. Korhonen

**Affiliations:** ^1^ NeuroMuscular Research Center, Faculty of Sport and Health Sciences, University of Jyväskylä, Jyväskylä, Finland; ^2^ Gerontology Research Center (GEREC), Faculty of Sport and Health Sciences, University of Jyväskylä, Jyväskylä, Finland; ^3^ Folkhälsan Research Center, Helsinki, Finland; ^4^ Faculty of Sport and Health Sciences, University of Jyväskylä, Jyväskylä, Finland; ^5^ Shanghai Jiao Tong University, Shanghai, China

**Keywords:** muscle, ageing, exercise, resistance training, fat mass, lean mass

## Abstract

**Introduction:**

Aging involves many physiological processes that lead to decreases in muscle mass and increases in fat mass. While regular exercise can counteract such negative body composition outcomes, masters athletes maintain high levels of exercise throughout their lives. This provides a unique model to assess the impact of inherent aging. The present study compared lean mass and fat mass in young and masters athletes from different sports to age-matched non-athletic individuals.

**Methods:**

Participants included young (20–39 years, *n* = 109) and older (70–89 years, *n* = 147) competitive male athletes, and 147 healthy age-matched controls (young = 53, older = 94 males). Athletes were separated into strength (e.g., weightlifters, powerlifters), sprint (e.g., sprint runners, jumpers) and endurance (e.g., long-distance runners, cross-country skiers) athletic disciplines. Body composition was assessed by dual-energy x-ray absorptiometry (DXA). Upper and lower limb lean mass was combined for appendicular lean mass as well as appendicular lean mass index (ALMI; kg/m^2^). Individuals’ scores were assessed against established cut-offs for low muscle mass, obesity, and sarcopenic obesity to determine prevalence in each group.

**Results:**

ALMI was greater in young strength (0.81–2.36 kg/m^2^, ∼15% and 1.24–2.74 kg/m^2^, ∼19%) and sprint (95% CI = 0.51–1.61 kg/m^2^, ∼11% and 0.96–1.97 kg/m^2^, ∼15%) athletes than in endurance and controls, respectively (all *P* < 0.001). In masters athletes, only strength athletes had greater ALMI than endurance athletes, but both older strength and sprint athletes had greater ALMI than older controls (0.42–1.27 kg/m^2^, ∼9% and 0.73–1.67 kg/m^2^, ∼13%, respectively, both *P* < 0.001). Fat mass was significantly lower in sprint and endurance athletes compared to strength athletes and controls in both age-groups. Sarcopenic obesity was identified in one young (2%) and eighteen (19%) older controls, while only two older endurance athletes (3%) and one older strength athlete (2%) were identified.

**Discussion:**

Lifelong competitive sport participation leads to lower prevalence of sarcopenic obesity than a recreationally active lifestyle. This is achieved in strength athletes by emphasizing muscle mass, while sprint and endurance athletes demonstrate low fat mass levels. However, all older athlete groups showed higher fat mass than the young groups, suggesting that exercise alone may not be sufficient to manage fat mass.

## 1. Introduction

Skeletal muscle and adipose tissue are involved in complex mechanical and biochemical interactions that contribute to maintaining a healthy cellular environment in several other tissues (1). Aging involves many physiological processes that lead to a decrease in muscle mass and an increase in fat mass that ultimately influences multiple aspects of health. Deteriorations in muscle mass are estimated to be ∼0.5% annually from the fifth decade onwards (2) while fat mass accrual is accelerated in middle-age with depots being re-distributed to the abdomen (3). Such body composition changes increase the risk of sarcopenia (i.e., muscle loss to the extent that function is compromised), metabolic ill-health and frailty later in life, particularly if the initial level during young adulthood is unfavorable. Nevertheless, understanding of the impact of aging *per se* on loss of muscle and increase in fat mass is complicated by the parallel decline in physical activity levels with increasing age in the general population (4, 5). Thus, lifelong exercisers who maintain high levels of physical activity can provide valuable insight about the efficacy of exercise to minimize deterioration in body structures and functions associated with typical aging (6). One such group is masters athletes, who maintain activity through their training regimes in order to compete to the best of their ability.

Distinctive features of various athletic disciplines include predominant training using high-load resistance training for strength athletes, such as weightlifters and throwers, high-impact and short-duration bursts of intense activity in sprint and jumper athletes, while endurance athletes (e.g., runners) complete high volumes of lower-intensity exercise. Such inherent differences in the training regimes of these athletes from different disciplines may have important consequences for both muscle and fat mass (i.e., body composition) maintenance during aging. Cross-sectional examination of muscle mass in masters athletes observed greater levels in athletes engaged in strength-/power-oriented sports compared with endurance athletes and controls (7–9). However, endurance-trained masters athletes and non-trained counterparts tend to have comparable muscle mass (7, 10).

One important outcome from scientific study of aging and exercise-enhanced maintenance of physical function, muscle and fat mass is the development of evidence-based practice. Studies have attempted to determine the efficacy of various exercise interventions on muscle and fat mass during older age (11, 12). While such studies provide evidence of adaptability of different aged person to exercise, it does not specifically address the issue of aging and how exercise may modify the trajectory of aging-induced changes in muscle and fat mass. This again provides justification for use of masters athletes as a model, given that they have trained in a specific manner for several decades.

When considering unfavorable changes in age-related body composition, an area that has challenged clinical identification is the use of body mass index (BMI), since simultaneous loss in muscle mass is hidden by (equivalent) increases in fat mass. Such a phenomenon has been recently termed sarcopenic obesity, and it is defined as the co-existence of obesity and both low muscle mass and low muscle function [i.e., sarcopenia (13)]. It is important to remember that sarcopenic obesity can occur at any age, and that excess fat and low muscle mass may act synergistically to influence daily physical activity/energy expenditure and increase the risk of cardiovascular comorbidities across adulthood and subsequently premature mortality (14).

The present study was able to examine total and regional fat mass as well as appendicular lean mass using DXA of a unique cohort young (20–39 years) and older (70–89 years) athletes from different sports, whose training and competition involves distinctly different practices, as well as healthy age-matched adults in order to identify the prevalence of sarcopenic obesity. It was hypothesized that strength athletes would have higher levels of muscle mass compared to endurance athletes and non-athletic individuals in both age-groups, while masters athletes from all athletic disciplines would have lower fat mass than non-athletic individuals for the older age-groups.

## 2. Materials and methods

In this cross-sectional study, data from the Athletes Aging Study (ATHLAS) including 109 young (20–39 years) and 147 older (70–89 years) male athletes from different sports was used. The athletes were recruited from among the members of Finnish sports organizations. On three occasions, the measurements were performed during competitions held in Jyväskylä. Based on the disciplines in which they competed, the athletes were separated into strength-trained (weightlifters, powerlifters, throwers), sprint-trained (jumpers, sprinters, hurdlers) and endurance-trained (long-distance runners, orienteers, cross-country skiers) groups. Age-matched healthy male controls were selected from the CALEX-family study carried out in the same laboratory [described in (15)]. Although some of the controls engaged in casual sport activities, none were involved in structured or competitive exercise training. Thus, eight groups in total were included to the study.

All participants were informed of the study’s details and provided written consent prior to their participation. The study was approved by the ethical committees of the Central Finland Health Care District (ATHLAS, Memo 4U/2012; CALEX, Memo 22/8/2008 and 5/2009) and was conducted in accordance with the principles of the Declaration of Helsinki.

Height and body mass were measured by standard methods using calibrated scales and body mass index (BMI) calculated as (kg/m^2^). Body composition parameters were measured by dual-energy x-ray absorptiometry (DXA, LUNAR Prodigy, GE Healthcare, Madison, WI, USA) whole-body scanning. Standard procedures recommended by the device manufacturer were followed. Participants lay supine on the device’s bed with the arms at the side and legs secured by strapping. From software-generated regions of interest, lean mass of the upper and lower limbs were obtained and combined to provide appendicular lean mass (ALM) estimates. Appendicular lean mass index (ALMI) was derived by dividing the individual’s ALM by the height-squared (kg/m^2^). Further, whole-body fat mass was obtained as well as android and gynoid region fat mass. Low muscle mass was defined as ALM < 20 kg, ALMI < 7.0 kg/m^2^ and obesity as >25%, respectively (16, 17) to identify individuals fulfilling the criteria of low muscle mass, obesity, and sarcopenic obesity.

As part of the ATHLAS and CALEX data collection, the years of systematic training and current training hours and number of training sessions per week were documented by a questionnaire. Additionally, the questionnaire included questions on participants’ known diseases and medication use. Descriptive characteristics of the participants are shown in Table 1.

**Table 1:** Subject characteristics of both age-groups separated by athletic discipline and non-training controls.

	Y Sprint (*n* = 41)	Y End (*n* = 35)	Y Strength (*n* = 33)	Y Con (*n* = 53)	O Sprint (*n* = 41)	O End (*n* = 59)	O Strength (*n* = 47)	O Con (*n* = 94)
Age (years)	28 ± 6	30 ± 5	30 ± 5	27 ± 5	77 ± 4	78 ± 5	77 ± 5	77 ± 5
Height (cm)	180 ± 5	180 ± 7	178 ± 7	180 ± 6	172 ± 5	172 ± 6	172 ± 8	171 ± 6
Body mass (kg)	79 ± 7	73 ± 9*	92 ± 14^§§,‡‡,^*	81 ± 14	71 ± 7*	71 ± 8**	84 ± 14^‡‡,§§^	77 ± 10
BMI (kg/m^2^)	24 ± 2^‡^	22 ± 2*	29 ± 4^‡‡,§§,^**	25 ± 4	24 ± 2**	24 ± 2**	28 ± 4^‡‡,§§,^*	26 ± 3
Number of training years^a^	17.5 ± 7.3*	15.8 ± 7.3	13.0 ± 6.4	10.1 ± 9.4	48.7 ± 18.0	49.9 ± 15.0*	36.6 ± 19.9^‡^	31.7 ± 25.4
Typical training frequency (times per week)	8.6 ± 2.9**	8.1 ± 4.1*	5.5 ± 2.9§	5.3 ± 2.7	4.0 ± 1.8	5.3 ± 2.6	4.6 ± 2.5	4.3 ± 2.9
Typical training duration (hours per week)	12.6 ± 5.7**	10.8 ± 5.1*	7.9 ± 3.9*§	5.0 ± 3.9	4.8 ± 3.0	6.7 ± 5.1*	7.5 ± 3.6**^,§^	4.0 ± 3.7

BMI, body mass index; End, endurance trained; Con, non-training control; Y, young; O, older.

Statistical analyses reported in the table refer to age-matched comparisons only.

^a^ The athletes documented sport-specific training while the age-matched controls documented low- to moderate-intensity physical activity, such as walking, cycling, gardening etc.

* *P* < 0.05.

** *P* < 0.001 compared to non-training controls.

^‡^ *P* < 0.05.

^‡‡^ *P* < 0.001 compared to endurance athletes.

^§^ *P* < 0.05.

^§§^ *P* < 0.001 compared to sprint athletes.

### 2.1. Statistical analyses

Means, standard deviations (SD) and 95% confidence intervals (95% CI) were calculated using standard methods. Normal distribution was assessed by the Shapiro-Wilk test and log transformation was used where variables were non-normally distributed within each group. To assess potential between-group differences (1) between different athletic disciplines and controls of the same age-group, and (2) between the two age-groups of the same athletic discipline/control), Welch’s test a modified one-way analysis of variance (ANOVA) that accommodates unequal group variances was used with all 8 groups included in the analyses. If a significant F-value was observed, post-hoc comparisons were explored using Tamhane’s T2 test. Alpha was set at 0.05. All analyses were performed using SPSS version 26 (IBM statistics, New York, USA).

## 3. Results

### 3.1. Subject characteristics

**Figure 1:**
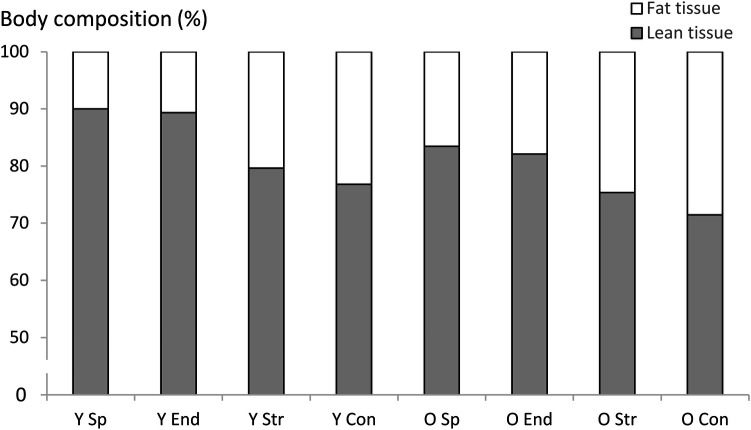
Body composition represented as the contribution of lean and fat tissue mass to overall body mass in each group. Y Sp = young sprint athletes, Y End = young endurance athletes, Y Str = young strength athletes, Y Con = young controls, O Sp = older sprint athletes, O End = older endurance athletes, O Str = older strength athletes, O Con = older controls.

Significant Welch test values were observed for age (Adjusted F_7,143_ = 625, *P* < 0.001), height (Adjusted F_7,148_ = 25.8, *P* < 0.001), body mass (Adjusted F_7,149_ = 17.1, *P* < 0.001) and BMI (Adjusted F_7,149_ = 24.7, *P* < 0.001), where post-hoc tests revealed that older adult groups were shorter than young adult groups, older sprint athletes had lower body mass than young sprint athletes, and older endurance athletes had greater BMI than their younger peers (Table 1). Within each age-group, young strength athletes had greater body mass than the other young groups and older strength athletes had greater body mass than older sprint athletes and endurance athletes. Both young and older endurance athletes had lower body mass than their age-matched non-athletic controls, while older sprint athletes also had lower body mass than older non-athletic controls. For BMI in both age groups, strength athletes had the largest BMI and endurance and sprint athletes had lower BMI than the non-athletic controls (Table 1). Figure 1 describes the body composition, i.e., relative contribution of lean and fat tissue to overall body mass, in each group.

### 3.2. Lean mass

**Figure 2:**
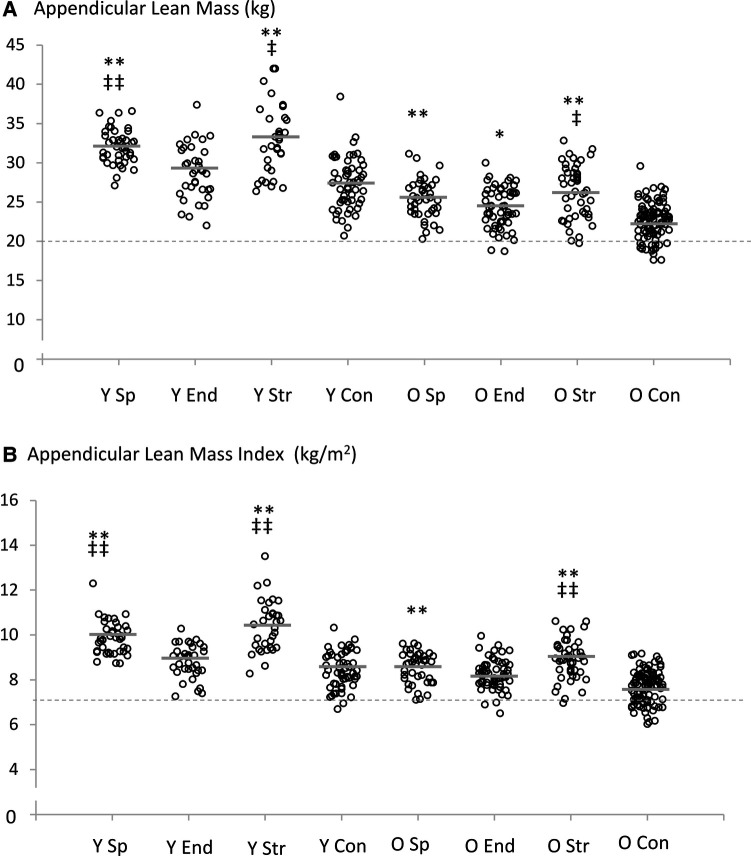
Individual and mean (grey lines) appendicular lean mass (**A**) and appendicular lean mass index (**B**) values for all groups. Statistical comparisons were made between the four groups within each specific age-range (20–39 years and 70–89 years). The dashed line shows the sarcopenia threshold according to Cruz-Jentoft et al. (16). Statistical analyses reported in the table refer to age-matched comparisons only; *P < 0.05, **P < 0.001 compared to non-athletic controls. ^‡^*P* < 0.05, ^‡‡^*P* < 0.001 compared to endurance athletes. Y Sp = young sprint athletes, Y End = young endurance athletes, Y Str = young strength athletes, Y Con = young controls, O Sp = older sprint athletes, O End = older endurance athletes, O Str = older strength athletes, O Con = older controls.

DXA data showed significant Welch test values for all lean mass outcome measures (Adjusted F_7,144–147 _= 53–90, *P* < 0.001). Strength athletes in both age-groups had greater appendicular lean mass compared to non-athletic controls (young: 95% CI = 2.60–8.41 kg, *P* < 0.001, older: 95% CI = 1.97–5.53 kg, *P* < 0.001) and also compared to endurance athletes (young: 95% CI = 0.98–7.22 kg, *P* = 0.002, older: 95% CI = 1.92–4.01 kg, *P* = 0.018). Young sprint athletes had greater appendicular lean mass compared to young endurance athletes (95% CI = 1.06–5.51 kg, *P* < 0.001) but no differences were observed in the older age-group (Table 2). Also, older endurance athletes demonstrated significantly greater appendicular lean mass than older non-athletic controls (95% CI = 0.35–2.95 kg, *P* = 0.003) (Figure 2A).

**Table 2:** Lean and fat mass, as well as fat percentage and android:gynoid ratio (mean ± SD) in each group assessed by dual-energy x-ray absorptiometry.

	Y Sprint (*n* = 41)	Y End (*n* = 35)	Y Strength (*n* = 33)	Y Con (*n* = 53)	O Sprint (*n* = 41)	O End (*n* = 59)	O Strength (*n* = 47)	O Con (*n* = 94)
Lower limb lean mass (kg)	23.0 ± 1.6**^,‡^	21.4 ± 2.4	23.0 ± 3.0**	20.0 ± 2.4	18.7 ± 1.8**	17.8 ± 1.7**	18.9 ± 2.6**	16.5 ± 1.7
Upper limb lean mass (kg)	9.0 ± 1.0**^,‡‡^	7.3 ± 1.2	9.8 ± 1.6**^,‡‡^	7.3 ± 1.1	6.7 ± 0.9*	6.5 ± 0.9	7.4 ± 1.0**^,‡‡,§^	6.1 ± 0.8
ALM (kg)	32.0 ± 2.2**^,‡‡^	28.8 ± 3.5	32.9 ± 4.4**^,‡^	27.4 ± 3.3	25.4 ± 2.5**	24.2 ± 2.5*	26.4 ± 3.4**^,‡^	22.6 ± 2.3
ALMI (kg/m^2^)	9.9 ± 0.7**^,‡‡^	8.8 ± 0.7	10.4 ± 1.1**^,‡‡^	8.4 ± 0.8	8.5 ± 0.7**	8.2 ± 0.7	8.9 ± 0.9**^,‡‡^	7.7 ± 0.7
Total fat mass (kg)	7.8 ± 4.3**	7.7 ± 4.4**	18.7 ± 8.2^‡‡,§§^	19.1 ± 10.2	11.5 ± 4.7**	12.4 ± 4.8**	20.5 ± 8.5^‡‡,§§^	21.6 ± 7.5
Fat (%)	10.0 ± 4.7**	10.7 ± 5.2**	20.4 ± 7.1^‡,§§^	23.2 ± 8.4	16.5 ± 5.8**	17.9 ± 5.8**	24.6 ± 7.2^‡‡,§§^	28.6 ± 7.1
Android fat mass (kg)	0.7 ± 0.4**	0.8 ± 0.5**	1.9 ± 1.0^‡‡,§§^	2.0 ± 1.3	1.4 ± 0.6**	1.5 ± 0.6**	2.5 ± 1.2^‡‡,§§^	2.5 ± 1.0
Gynoid fat mass (kg)	1.6 ± 0.8**	1.5 ± 0.8**	3.2 ± 1.2^‡‡,§§^	3.4 ± 1.5	1.8 ± 0.6**	1.9 ± 0.6**	2.7 ± 0.9^‡‡,§§^	3.1 ± 1.0
Android:gynoid ratio	0.4 ± 0.1**	0.5 ± 0.1	0.6 ± 0.1^§§^	0.6 ± 0.2	0.7 ± 0.2	0.8 ± 0.2	0.9 ± 0.3	0.8 ± 0.2

ALM, appendicular lean mass; ALMI, appendicular lean mass index; BMI, body mass index; End, endurance trained; Con, non-training control; Y, young; O, older.

Statistical analyses reported in the table refer to age-matched comparisons only.

* *P* < 0.05.

** *P* < 0.001 compared to non-training controls.

^‡^ *P* < 0.05.

^‡‡^ *P* < 0.001 compared to endurance athletes.

^§^ *P* < 0.05.

^§§^ *P* < 0.001 compared to sprint athletes.

For ALMI, significant differences were observed for both sprint and strength athletes compared to non-athletic controls in both age-groups (young: 95% CI = 0.96–1.97 kg/m^2^ and 1.24–2.74 kg/m^2^, older: 95% CI = 0.42–1.27 kg/m^2^ and 0.73–1.67 kg/m^2^, respectively, all *P* < 0.001) (Figure 2B). Strength athletes also demonstrated greater ALMI than endurance athletes in both age-groups (young: 95% CI = 0.81–2.36 kg/m^2^, *P* < 0.001, older: 95% CI = 0.17–1.15 kg/m^2^, *P* = 0.001) (Table 2). Young sprint athletes had greater ALMI than young endurance athletes (95% CI = 0.51–1.61 kg/m^2^, *P* < 0.001).

The individuals displaying appendicular lean mass below the proposed 20 kg cut-off for low muscle mass (16) for males were predominantly from the older non-athletic control group; fifteen (16%). Two (3%) older endurance athletes and one (2%) older strength athlete also displayed appendicular lean mass below 20 kg.

There were nineteen (20%) older non-athletic controls and even two (4%) young non-athletic controls displaying ALMI below the proposed low muscle mass cut-off of 7.0 kg/m^2^. There were also three (5%) older endurance athletes and one (2%) older strength athlete below this cut-off.

The pattern of lower lean mass between non-athletic individuals and all athletic disciplines was evident in leg lean mass, however, higher lean mass in strength athletes over other athletic disciplines was evident in upper limb lean mass, particularly in the older group (Table 2). Older strength athletes had higher upper limb lean mass (7.4 ± 1.0 kg) compared to sprint (6.7 ± 0.9 kg, *P* = 0.010) and endurance (6.5 ± 0.9 kg, *P* < 0.001) athletes. Young sprint athletes (9.0 ± 1.0 kg) had greater upper limb lean mass compared to endurance athletes (7.3 ± 1.2 kg, *P* < 0.001) and non-athletic controls (7.3 ± 1.1 kg, *P* < 0.001).

### 3.3. Fat mass

Significant Welch test values for all fat mass, fat percentage, and android:gynoid ratio measures were observed (Adjusted F_7,145–152_ = 33–64, *P* < 0.001). Post hoc tests revealed that sprint and endurance athletes had lower fat mass/percentage compared to non-athletic controls (*P* < 0.001), regardless of age-group (Table 2). Also, sprint and endurance athletes had lower fat mass/percentage compared to strength athletes (*P* < 0.001), regardless of age-group. However, the only statistically significant differences in android:gynoid ratio were observed between young sprint athletes and non-athletic controls as well as between young sprint and strength athletes (Table 2).

**Figure 3:**
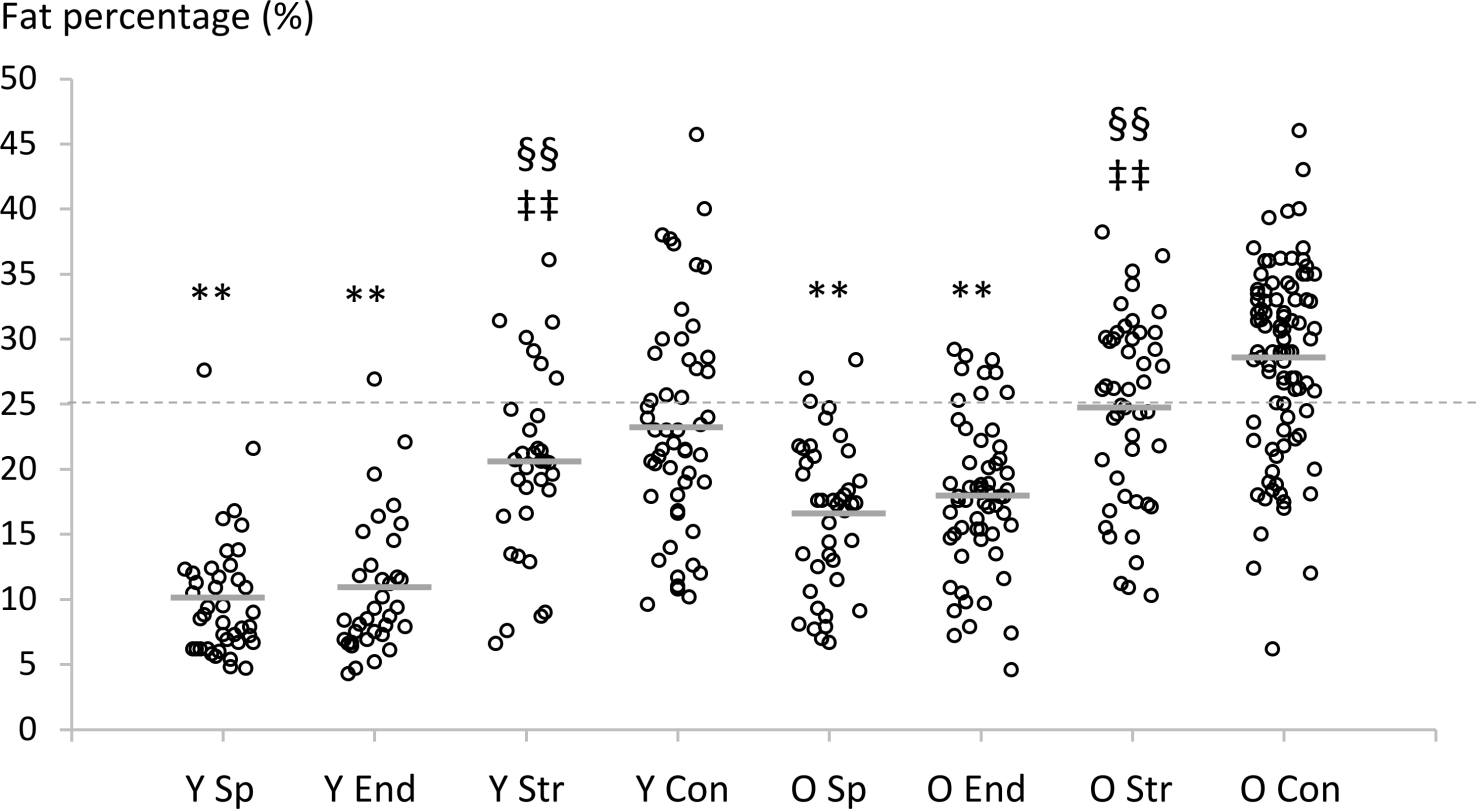
Individual and mean (grey lines) fat percentage values for all groups. Statistical comparisons were made between the four groups within each specific age-range (20–39 years and 70–89 years). The dashed line shows the obesity threshold according to the American Society of Bariatric Physicians (17). Statistical analyses reported in the table refer to age-matched comparisons only; *P < 0.05, **P < 0.001 compared to non-athletic controls. ^‡^*P* < 0.05, ^‡‡^*P* < 0.001 compared to endurance athletes, ^§^*P* < 0.05, ^§§^*P* < 0.001 compared to sprint athletes. Y Sp = young sprint athletes, Y End = young endurance athletes, Y Str = young strength athletes, Y Con = young controls, O Sp = older sprint athletes, O End = older endurance athletes, O Str = older strength athletes, O Con = older controls.

The individuals above the obesity threshold for fat percentage (>25%) according to the American Society of Bariatric Physicians (17) were predominantly from non-athletic control and strength training groups, regardless of age. There were nineteen (36%) identified from the young age-group and sixty-eight (72%) identified from the older non-athletic controls. In strength athletes, there were seven (21%) and twenty-four (51%) individuals identified in young and older age-groups, respectively. In sprint and endurance athletes, there were one (2%) and one (3%) individual in the young age-group, respectively, and three (7%) and nine (15%) individuals in the older age-group, respectively (Figure 3).

### 3.4. Sarcopenic obesity prevalence

The prevalence of sarcopenic obesity (i.e., the number of individuals demonstrating lean mass below the low muscle mass cut-off and fat percentage above the obesity cut-off) was identified in one young non-athletic control (2%). In the older groups, two endurance athletes (3%), 1 strength athlete (2%), and eighteen non-athletic controls (19%) were identified.

### 3.5. Age-group comparisons

Table 3 shows the 95% confidence intervals for the differences between age-groups along with the *P*-values from Tamhane’s T2 test. Upper and lower limb lean mass was significantly different between young and older age-groups, regardless of athletic discipline and in non-athletic controls. Fat mass, fat percentage, android fat mass and android:gynoid ratio was significantly different between young and older age-groups for sprint and endurance athletes. The only fat tissue outcome measure that was significantly different between young and older age-groups in strength athletes was android:gynoid ratio. In non-athletic controls, fat percentage, android fat mass and android:gynoid ratio was significantly different between young and older age-groups (Table 3).

**Table 3:** Post hoc comparisons (95% confidence intervals for the differences and *P*-values from tamhane’s T2 test) between different age-groups within each athletic disciplines and non-training controls in lean tissue and fat tissue variables.

	Young vs. Older	Young vs. Older	Young vs. Older	Young vs. Older
	Sprint athletes	Endurance athletes	Strength athletes	Non-training controls
Lower limb lean mass (kg)	3.09 to 5.46 (<0.001)	2.14 to 5.17 (<0.001)	1.99 to 6.20 (<0.001)	2.31 to 4.72 (<0.001)
Upper limb lean mass (kg)	1.68 to 3.06 (0.001)	0.06 to 1.64 (0.023)	1.40 to 3.43 (<0.001)	0.68 to 1.78 (<0.001)
ALM (kg)	4.98 to 8.32 (0.001)	2.32 to 6.70 (<0.001)	3.55 to 9.47 (<0.001)	3.09 to 6.41 (<0.001)
ALMI (kg/m^2^)	0.83 to 1.84 (<0.001)	0.83 to 1.08 (0.009)	0.73 to 2.27 (<0.001)	0.29 to 1.14 (<0.001)
Total fat mass (kg)	−6.91 to −0.49 (0.003)	−7.82 to −1.55 (<0.001)	−7.94 to −4.35 (1.000)	−7.65 to 2.68 (0.559)
Fat%	−10.31 to −2.78 (<0.001)	−10.92 to −3.39 (<0.001)	−9.50 to 0.99 (0.257)	−9.75 to −0.98 (0.005)
Android fat mass (kg)	−1.07 to −0.28 (<0.001)	−1.10 to −0.35 (<0.001)	−1.44 to 0.14 (0.451)	−1.22 to 0.08 (0.023)
Gynoid fat mass (kg)	−0.73 to 0.30 (0.723)	−0.89 to 0.14 (0.130)	−0.40 to 1.27 (0.999)	−0.38 to 1.10 (1.000)
Android:Gynoid ratio	−0.44 to −0.18 (<0.001)	−0.40 to −0.16 (<0.001)	−0.49 to −0.17 (<0.001)	−0.37 to −0.18 (<0.001)

ALM, appendicular lean mass; ALMI, appendicular lean mass index.

## 4. Discussion

Although the specificity of exercise in young adult athletes is well described, very little information exists on efficacy of lifelong exercise patterns to maintain healthy muscle mass and body fat beyond the age of 70, when the cumulative negative (age-related) changes in body composition often start to compromise clinical health and functioning. The present study provides new information of the accompaniment of systematic strength, sprint and endurance training on indices of body composition in young and older male athletes. We found that strength athletes had the highest appendicular lean mass (ALM) and appendicular lean mass index (ALMI) in both young and older age-groups, which supports the hypothesis regarding strength athletes having greater muscle mass than endurance and control groups. When assessing the upper- and lower-limbs separately, the differences between athletic disciplines in favor of strength and sprint groups was most apparent in upper limb lean mass, particularly in older age. The second hypothesis, regarding lower fat mass in athletes, was partly supported in that sprint and endurance athletes showed lower fat mass, but strength athletes did not differ from non-athletic controls. This was also observed in the young age-groups. In older non-athletic controls, as expected, the prevalence of low muscle mass (16%–20%), obesity (72%), and sarcopenic obesity (19%) was highest.

### 4.1. Lean mass

Although the prevalence of low muscle mass in the present study (e.g., 16%–20% in older non-athletic controls) may be deemed as lower than observed in cohort studies [e.g., ∼41% (18)], this may be due to individuals that are generally interested in health and well-being volunteering for our studies. Nevertheless, clear between-group differences were observed in the present study.

Studies in young athletes indicate that long-term participation in strength and power sports co-exist with marked muscular hypertrophy along with maximal strength and force-time characteristics while aerobic exercise induces specific cardiorespiratory benefits with limited effect on muscle size (19, 20). There is also some evidence from middle-aged and older athletes that athletic disciplines containing heavy resistance training regimes, which are typically employed by weightlifters and throwers, have greater muscle mass than endurance-trained and non-athletic individuals (7–10, 21). This was also observed in the present study, with perhaps the greatest differences observed for upper rather than lower limb lean mass (Table 2). Interestingly, endurance training was sufficient to demonstrate significant differences in leg lean mass compared to non-athletic controls at older age in the present study. Further, the prevalence of low muscle mass in our masters endurance athletes (3%–5%) was markedly different than non-athletic controls. At older age, the sprinters did not demonstrate significant differences in muscle mass compared to endurance athletes, whereas strength athletes did. One possible explanation for the separation of strength and sprint athletes in older age may be due to training habits of older sprinters, whose past and current training has consisted mainly of running practices with very little involvement in muscle-building heavy resistance training (22).

Skeletal muscle is an important tissue in homeostatic regulation. It is responsible for the majority of post-prandial glucose clearance (23), its energy utilizing during contraction likely influences insulin sensitivity hepatically and peripherally (24), and its release of myokines may be an important element in trans-organ cross-talk and ultimately health (1). Thus, it may be speculated that a greater tissue mass, especially one that is active, has an enhanced impact on whole-body metabolic health.

Force generation capacity (i.e., strength) is another important consideration in healthy aging. For instance, stair climbing ability has been shown to be limited by maximum strength, leading to alternative, and presumably less efficient, biomechanical strategies to complete the functional task (25). The ability to produce force is predicted primarily by muscle mass in young and older adults (26, 27), as well as voluntary activation level. Thus, lower muscle mass influencing force production may lead to mobility limitation and reduced physical activity (28), which may ultimately increase the risk of disability and reduce the ability to live an independent everyday life in older age (29). While muscle mass has been shown to be important for mobility, the most prominent indicators are maximum strength and power (30–32). This raises the possibility that muscle mass alone may not be a fully representative measure of physical performance in healthy older men. It is worth pointing out that none of the individuals identified with low muscle mass or as sarcopenic obese reported functional impairment at the time of testing, suggesting that they might not have reached the point where their deconditioning noticeably impacts daily functioning.

### 4.2. Fat mass

In the present study, strength athletes and non-athletic controls demonstrated similar fat mass characteristics regardless of the DXA-derived outcome measure. Fat percentage values of our strength athletes and non-athletic controls closely matched cohort reports of Chinese adults throughout the lifespan and were markedly lower than reports from USA population (33). They were also close to the mean 23% fat reported in Finnish middle-aged males (34). Young and older sprint and endurance athletes had significantly less fat mass in both absolute and relative scales compared to strength and control groups. The only exception was in the android:gynoid ratio where limited significant differences were observed between groups.

The magnitude of the differences in fat percentage between young and older athletes in the present study (approx. 5 percentage point higher is in-line with previous cross-sectional and longitudinal follow-up studies, showing 5–10 percentage point increases in athletes (35). Regardless of athletic discipline, such differences were observed suggesting a higher age-related inevitability of increased fat percentage. Using a similar approach in comparing athletic disciplines, future studies could tease out nuances in age-related fat percentage differences (i.e., fat increase versus muscle loss) to then develop better targeted training programs for aging individuals. However, it should be noted that the present study is a cross-sectional comparison and direct inferences of changes over time should not be made.

The prevalence of sarcopenic obesity in non-athletic controls of the present study (19%) is within the limits of previous estimates (36), although prevalence is difficult to determine fully given a lack of accepted definition. Nevertheless, up to 8-fold increased risk of metabolic syndrome in individuals classified as having sarcopenic obesity has been reported along with increased risk for other cardiovascular/metabolic diseases (14). Hence, prevention is of paramount importance. The present study has shown that sprint and endurance athletic disciplines are preferable for lower fat mass, android fat mass, and fat percentage in both young and older age. According to the cut-off proposed by the American Society of Bariatric Physicians (17), prevalence of obesity was low in these athletes (<15% compared to 72% in older non-athletic controls) and sarcopenic obesity in older age was only observed in two (3%) endurance athletes as opposed to eighteen (19%) non-athletic controls. Risk of death from cardiovascular disease and cancer is markedly increased in the bottom 20th percentile for fat mass (37). This would translate to increased risk for some young and older non-athletic control group subjects and also for some of the older strength athletes in the present study. The strength athletes naturally do not need to transport their body mass over a particular distance, and so body mass *per se* may not be a disadvantage to their sport. Additionally, in order to support muscle hypertrophy, strength athletes typically consume excess calories, which may have also inadvertently influenced fat mass accrual especially in more experienced athletes (38). However, from a health perspective, it would be beneficial for strength athletes to lower fat mass levels through a combination of aerobic activity and diet (as sprint and endurance athletes typically do) as a supplement to their performance-specific training.

Central obesity, visceral or abdominal depending on the assessment method, is thought to be particularly deleterious to cardiovascular and metabolic health. A preferable android:gynoid ratio is generally considered to be below 1 to reduce the risk of developing the cardiovascular/metabolic diseases detailed above. In the present study, average android:gynoid ratio values were below 1 in all groups, with the older groups being closest to this threshold. Thus, none of the groups in the present study displayed particularly regionalized fat distribution around the abdomen, highlighting the relatively good body composition of the subjects overall in the present study.

### 4.3. Strengths and weaknesses of the study

A clear strength of the present study is the relatively large sample of competitive athletes from different disciplines. In total, 256 athletes agreed to participate and were scanned for body composition to allow comparisons with a non-competitive but healthy age-matched cohort. Directly assessing body composition via DXA circumnavigates some of the inherent weaknesses of investigating low muscle mass, obesity, and sarcopenic obesity using BMI or waist/hip circumference methods. The present study has, thus, likely identified prevalence of low muscle mass, obesity, and sarcopenic obesity that can be used for comparative purposes between populations.

One weakness in the present study is that the study was partially conducted during competition conditions that precluded us to carry out other tests often used in sarcopenic assessment (e.g., muscle strength, chair rise test, walking speed). Thus, by strict definition, neither sarcopenia nor consequently sarcopenic obesity can be fully identified. Second, the use of DXA does not allow accurate assessment of intra-abdominal fat nor possible fat infiltration within the muscle that has been shown to occur particularly in sedentary older adults. On the other hand, DXA measures may under-represent the health value of exercise in sprint and endurance athletes who likely have low intra-abdominal fat. Another weakness of the present study is that there were only males examined. Some evidence suggests that aging and physical exercise habits may impact on muscle mass and fat mass at a varying rate between the sexes (39). Therefore, it remains unknown as to how aging and age-related physiological processes (including menopause) affects comparisons between competitive females from different disciplines and non-athletic individuals. Future studies should address the imbalance in knowledge regarding the effects of lifelong training/athletic competition between the sexes. Finally, potentially important confounders were not able to be accounted for in the present study. Covariates such as nutrition, precise training volume/intensity, nor injury/illness could not be included into statistical analyses.

## 5. Conclusion

Competitive sport participation throughout adult life leads to a considerably lower prevalence of sarcopenic obesity than a recreationally active lifestyle. This appears to be achieved in strength athletes by emphasizing muscle mass, while sprint and endurance athletes demonstrate low levels of fat mass. However, even lifelong athletes showed higher fat mass than young athletes, regardless of athletic discipline. This suggests that other interventions than just exercise (e.g., diet) may be necessary to manage fat mass in order to maintain a more optimal body composition in older age, which would be particularly important for those approaching the obesity threshold, as shown in the strength athletes and non-athletic controls.

## Data Availability

The raw data supporting the conclusions of this article will be made available by the authors, without undue reservation.
